# The Lake Victoria island intervention study on worms and allergy-related diseases (LaVIISWA): study protocol for a randomised controlled trial

**DOI:** 10.1186/s13063-015-0702-5

**Published:** 2015-04-23

**Authors:** Margaret Nampijja, Emily L Webb, James Kaweesa, Robert Kizindo, Milly Namutebi, Esther Nakazibwe, Gloria Oduru, Prossy Kabuubi, Joyce Kabagenyi, Dennison Kizito, Lawrence Muhangi, Mirriam Akello, Jaco J Verweij, Barbara Nerima, Edridah Tukahebwa, Alison M Elliott, for the LaVIISWA trial team

**Affiliations:** MRC/UVRI Uganda Research Unit on AIDS, PO Box 49, Entebbe, Uganda; London School of Hygiene and Tropical Medicine, Keppel Street, London, WC1E 7HT UK; Vector Control Division, Ministry of Health, PO Box 1661, Kampala, Uganda; Entebbe Hospital, PO Box 29, Entebbe, Uganda; Laboratory for Medical Microbiology and Immunology, St Elisabeth Hospital, Postbus 90151, 5000 LC Tilburg, the Netherlands; Uganda Virus Research Institute, PO Box 49, Entebbe, Uganda

**Keywords:** Helminths, Anthelminthic treatment, Allergy, Atopy, Wheeze, Cluster-randomised trial

## Abstract

**Background:**

The Hygiene Hypothesis proposes that infection exposure protects against inflammatory conditions. Helminths possess allergen-like molecules and may specifically modulate allergy-related immunological pathways to inhibit responses which protect against them. Mass drug administration is recommended for helminth-endemic communities to control helminth-induced pathology, but may also result in increased rates of inflammation-mediated diseases in resource-poor settings. Immunological studies integrated with implementation of helminth control measures may elucidate how helminth elimination contributes to ongoing epidemics of inflammatory diseases. We present the design of the Lake Victoria Island Intervention Study on Worms and Allergy-related diseases (LaVIISWA), a cluster-randomised trial evaluating the risks and benefits of intensive versus standard anthelminthic treatment for allergy-related diseases and other health outcomes.

**Methods/Design:**

The setting is comprised of island fishing communities in Mukono district, Uganda. Twenty-six communities have been randomised in a 1:1 ratio to receive standard or intensive anthelminthic intervention for a three-year period. Baseline characteristics were collected immediately prior to intervention rollout, commenced in February 2013. Primary outcomes are reported wheeze in the past 12 months and atopy (skin prick test response and allergen-specific immunoglobulin (asIg) E concentration). Secondary outcomes are visible flexural dermatitis, helminth infections, haemoglobin, growth parameters, hepatosplenomegaly, and responses to vaccine antigens. The trial provides a platform for in-depth analysis of clinical and immunological consequences of the contrasting interventions.

**Discussion:**

The baseline survey has been completed successfully in a challenging environment. Baseline characteristics were balanced between trial arms. Prevalence of *Schistosoma mansoni*, hookworm, *Strongyloides stercoralis* and *Trichuris trichiura* was 52%, 23%, 13%, and 12%, respectively; 31% of *Schistosoma mansoni* infections were heavy (>400 eggs/gram). The prevalence of reported wheeze and positive skin prick test to any allergen was 5% and 20%, respectively. Respectively, 77% and 87% of participants had *Dermatophagoides*- and German cockroach-specific IgE above 0.35 kUA/L. These characteristics suggest that the LaVIISWA study will provide an excellent framework for investigating beneficial and detrimental effects of worms and their treatment, and the mechanisms of such effects.

**Trial registration:**

This trial was registered with Current Controlled Trials (identifier: ISRCTN47196031) on 7 September 2012.

**Electronic supplementary material:**

The online version of this article (doi:10.1186/s13063-015-0702-5) contains supplementary material, which is available to authorized users.

## Background

Allergy-related conditions represent a huge global health burden [1-4], with the majority of asthma and eczema cases associated with atopy (elevated allergen-specific immunoglobulin (Ig) E or skin prick test (SPT) responses to allergens). Prevalence of these conditions increased dramatically in affluent and middle-income countries during the 20th century [5-7], with asthma now affecting about 300 million people [3], and eczema affecting 5 to 20% of children [2]. Asthma mortality depends on both prevalence and quality of care [6,8]: currently, asthma prevalence is increasing more rapidly and asthma severity is greater in low- and middle-income countries [9]. As the low-income countries of sub-Saharan Africa enter the epidemiological transition, they are ill-equipped to encounter such an epidemic.

Globally, around one billion people may be infected with helminths [10], which cause many subtle and some severe morbidities [10,11]. Major initiatives for mass anthelminthic treatment have commenced in the last decade, with some success [12]. However, the Hygiene Hypothesis proposes that exposure to infection protects against inflammatory conditions, including allergy-related diseases. If this is correct, effective de-worming will bring risks as well as benefits.

Untreated, helminth parasites survive for decades in their mammalian hosts and, to do so, must evade or modulate the host immune response. Evidence from animal models, and from *in vitro* studies on human samples, suggests that helminths can modulate the immune response not only to themselves, but also to unrelated pathogens, antigens, immunogens, and allergens [13,14]. Helminth infections may increase susceptibility to microbial pathogens and reduce vaccine efficacy [15]. Helminths may have particular effects relating to allergic inflammation because they contain a range of molecules homologous to known allergens, but absent from mammals, or markedly different from mammalian homologues. These induce IgE responses in mammalian hosts, and there is strong evidence that this pathway is involved in protective immunity against helminths [16]. Modulation of this atopic pathway is therefore likely to be particularly important for helminth survival, while concomitantly protecting against allergic disease [17].

In cross-sectional studies, consistent inverse associations have been observed between helminths and atopy, and between hookworm and asthma [18]. Possible explanations are either that helminths protect against asthma and atopy, or that people genetically predisposed to asthma and atopy are resistant to helminths, with the latter hypothesis supported by reports that certain genotypes are associated with both risk of atopy and resistance to helminths [19,20]. Intervention studies are thus key to elucidating the relationship between helminths and allergy, but results are conflicting. Initial studies in Venezuela [21], Gabon [22], and Vietnam [23] suggested that atopy, as assessed by SPT positivity, increased following regular anthelminthic treatment. However, a large cluster-randomised trial among schools in rural Ecuador showed no effect of two-monthly albendazole for one year on SPT responses [24]. One possible explanation for these different findings is differing prevalent helminth species. No major trial has yet investigated the effects of treatment of schistosomiasis on asthma, eczema, and atopy, although schistosomiasis has shown a strong inverse association with atopy in observational studies [25].

Apart from effects on allergy, interventions against helminths, particularly schistosomiasis, may have important benefits for anaemia, growth, hepatosplenic morbidity, vaccine responses, and cognitive development [11,12,26], but the community-level effectiveness of mass anthelminthic treatment for some of these outcomes [27,28] is not known.

As mass drug administration is increasingly advocated and implemented, there is a window of opportunity to investigate whether, and how, helminths protect against allergy-related diseases in an epidemiological setting that no longer exists in high-income countries, and to assess the risks and benefits of mass anthelminthic treatment. The Lake Victoria Island Intervention Study on Worms and Allergy-related diseases (LaVIISWA; Current Controlled Trials identifier: ISRCTN47196031) was designed to address these questions. We summarise the LaVIISWA study protocol, describe baseline characteristics of the study population, and discuss experiences from initiation of the trial.

## Methods/Design

### Study setting

**Figure 1:**
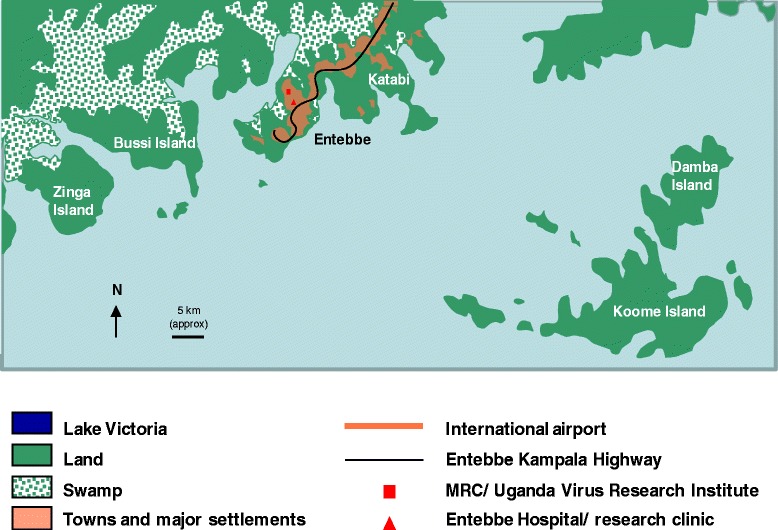
Location of the study area.

The LaVIISWA study is being conducted in the Lake Victoria islands of Koome sub-county in Mukono district, Uganda, which has an approximate population of 16,000 people (Figure 1). The area is remote, being accessible in two to three hours from Entebbe by motor powered canoe. The national programme of regular mass anthelminthic treatment is hampered by the cost of reaching these communities, and by inadequate drug supply.

**Figure 2:**
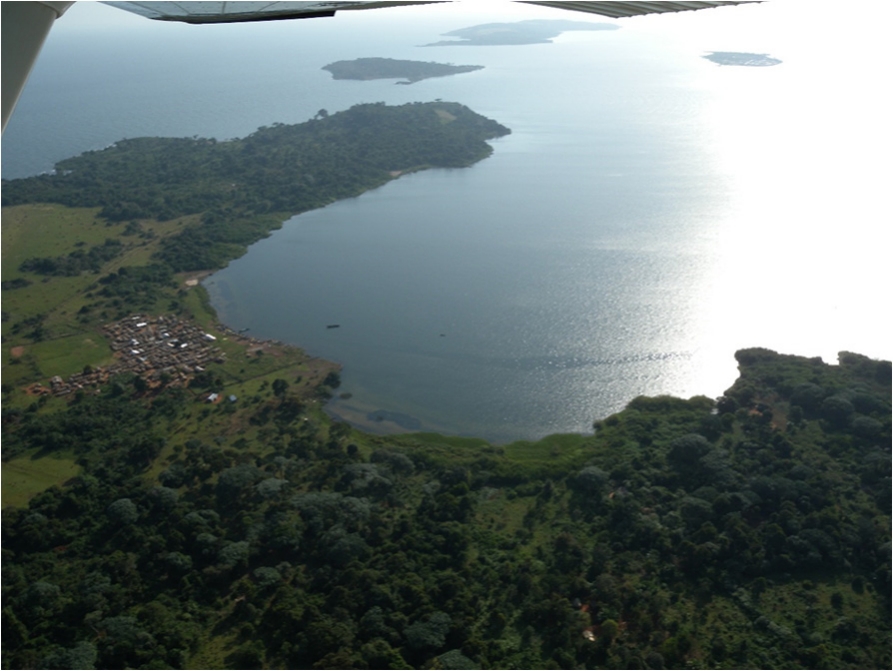
Aerial photograph of the study setting (courtesy of Professor Russell Stothard).

**Figure 3:**
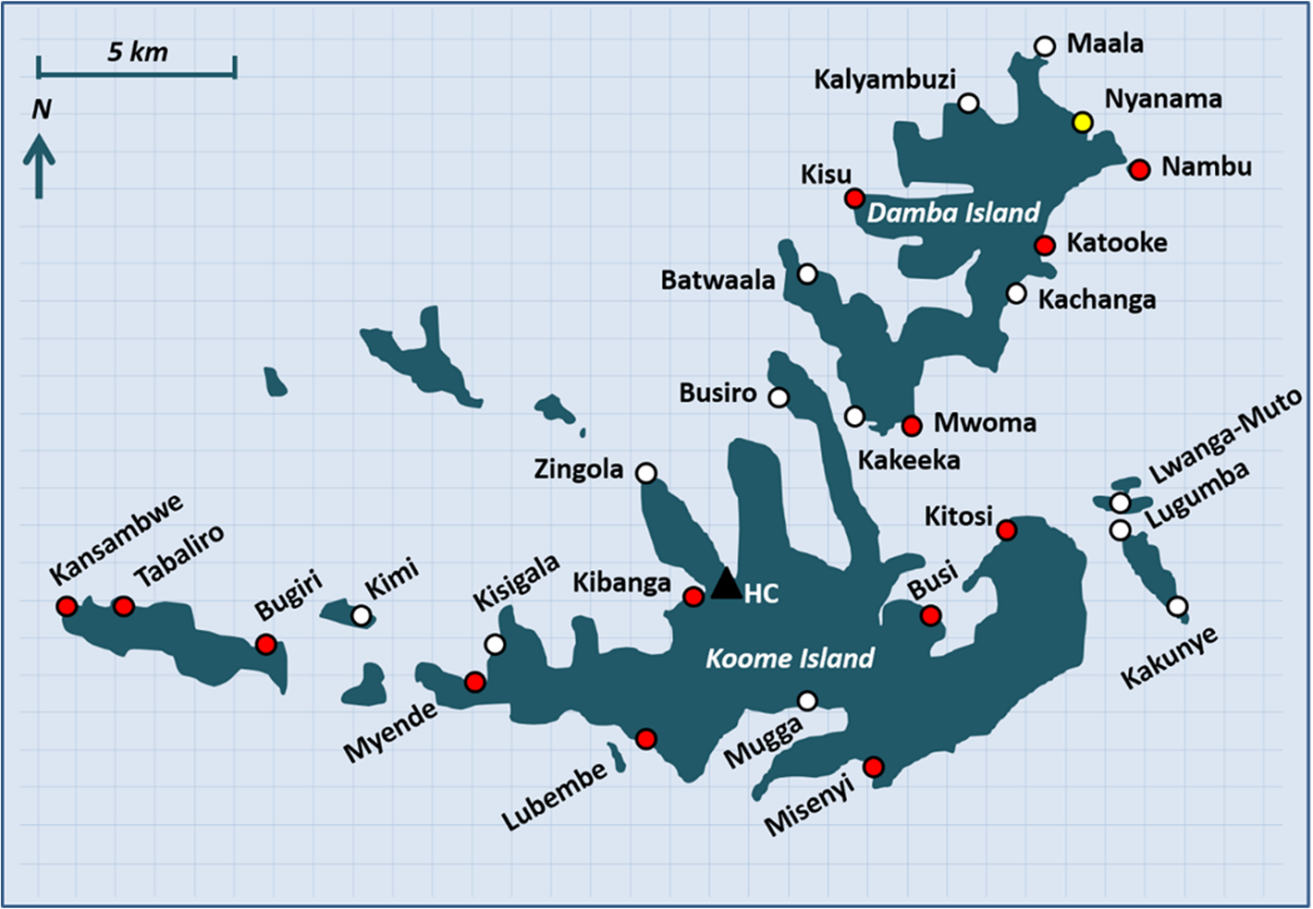
Study villages (clusters), Koome sub-county, Mukono district, Uganda. Villages receiving standard intervention indicated in white, villages receiving intensive intervention indicated in red, pilot village indicated in yellow, location of the main health centre (HC) indicated as a black triangle.

The island communities consist of well-defined, geographically separated villages, each governed by a single administrative committee, and mainly located on the islands’ shores (Figure 2). For this study, fishing villages only (not inland villages) are included. There are 27 fishing villages in Koome sub-county (Figure 3) and all are taking part in the study.

### Study design

The LaVIISWA study is an open cluster-randomised trial of intensive versus standard anthelminthic treatment. Randomisation at the individual or household level would lead to treatment effects being diluted by re-infection from untreated neighbours, whereas interventions provided to the whole village are expected to result in a reduction in transmission within the whole village.

Fishing villages are the units of randomisation. One of the villages was randomly selected as a pilot village in order to optimise procedures, with the remaining 26 villages participating in the full trial. Clusters receive either intensive or standard anthelminthic treatment over a three-year period. A baseline household survey was conducted immediately prior to the first treatment round; outcomes will be assessed at the end of the three years using a second household survey.

A small number of the villages comprise more than one cluster of households and many comprise a cluster of households located directly on the lake shore and scattered inland households. In such villages, all households receive the allocated intervention, but the sampling frame for the two household surveys only includes households in the main cluster of households. This ‘fried egg’ design creates buffer zones between clusters, reduces risk of contamination, and minimises variability in cluster characteristics.

### Information and consent

This project is being conducted in collaboration with the Vector Control Division (VCD) at the Uganda Ministry of Health. Prior to the start of the trial, the proposed protocol was presented to, and consultations held with, district leaders. Consultations were also held with health service providers on the islands, and collaborations were established to facilitate the work and coordinate with outreach activities to remote communities. Village health teams (VHTs) are working with the research team during surveys and for drug distribution activities. Meetings were held in each of the villages to explain the work and answer questions. The local council committees in each village were asked for written permission for the trial to be conducted there.

Written individual consent for participation in the intervention was not sought since this is a community-based intervention and the minimum provision meets the standard recommended by the VCD. Although individuals are encouraged to participate in the intervention programme allocated to their village, they may refuse treatment at any time, and this was made clear at the meetings and continues to be emphasised during treatment distribution. Individual written informed consent (for adults aged ≥18 years and emancipated minors, and for children by a parent or guardian) and assent (for children aged 8 to 17 years) are sought for survey participation.

### Interventions

The standard intervention (as recommended by the VCD) comprises home-delivered single-dose albendazole (400 mg) given twice-yearly to all community members aged ≥1 year, and annual praziquantel treatment (approximately 40 mg/kg, estimated by height pole) to all individuals measuring ≥94 cm (the standard height pole range) [29]. This height range excludes many pre-school children [30]. Individuals who are sick are excluded.

The intensive intervention comprises home-delivered triple-dose albendazole and single-dose praziquantel, both given quarter-yearly to all community members aged one year or older. For praziquantel administration to young children, the extended height pole (≥60 cm) is used, with tablets crushed and given in juice [31] because a paediatric formulation for praziquantel is not yet available. For albendazole, a three-day course of 400 mg daily is given, in order to provide more effective treatment for *Trichuris trichiura* [32,33]. The first dose is directly observed, and the subsequent two doses provided. Pregnant women are given only single-dose albendazole; this is considered acceptable in pregnancy by the World Health Organization (WHO) [34], but multiple doses might lead to accumulation of higher drug levels, with possible adverse consequences [34-36].

The intervention is not blinded. Treatments for both arms of the trial are provided by the research programme, supervised by research team members in collaboration with the VCD, and distributed by VHTs. The VHT distributors document uptake against household registers, recording individual participation or refusal of treatment and adverse effects.

### Randomisation

A public randomisation ceremony was conducted at a school near the sub-county headquarters on 15 August 2012, attended by representatives from each community and sub-county leaders. The randomisation ceremony had three stages. In stage one, a pilot village (to receive standard intervention) was randomly selected, from a shortlist of eight villages categorised as suitable for piloting activities on the basis of size and accessibility, by a community member who drew a numbered ball from an opaque bag. In stage two, restricted randomisation was then used to allocate the remaining 26 villages in a 1:1 ratio to the trial arms, while ensuring balance on village size, distance to Koome health centre (the main health centre on the islands), and previous helminth treatment coverage. Village size was approximated by the number of households, estimated using information from local council leaders. Villages were grouped into four categories (>1,000 households, 600 to 1,000 households, 300 to 599 households, or <300 households). Distances from each village centre to Koome health centre were calculated from GPS coordinates. Previous helminth treatment coverage was defined as the estimated number of people treated with praziquantel (based on national programme records) divided by the estimated village population. The following restrictions were applied:

1. Village size: number of villages within each size category must not differ between the two arms by more than one.
2. Distance to Koome health centre: difference between the two arms in mean distance must be less than 0.25 km.
3. Previous helminth treatment coverage: the difference between the two arms in mean coverage (estimated proportion treated) must be lower than 0.01.

For each of the 8 possible pilot villages, a random sample of 1,000 possible allocations of the remaining 26 villages to the two trial arms, satisfying the restrictions, was pre-prepared by the study statistician. Possible allocations, with 13 villages assigned to group 0 and 13 villages assigned to group 1, were numbered from 000 to 999. In stage two, three balls that together gave a number between 000 and 999 were randomly selected by community members, and the corresponding allocation from the list was used to assign each village to group 0 or group 1. Finally, in stage three, a ball was selected at random by the sub-county chairman to determine which group (1 or 0) would receive the intensive and which would receive the standard intervention.

### Baseline and three-year household surveys

A baseline survey was conducted in each cluster immediately prior to the first treatment round, allowing us to assess the comparability of villages between the two arms and the trial analysis to be adjusted for any baseline imbalances; this information could not be used for the restricted randomisation, since for logistical reasons it was necessary to initiate the intervention in each cluster immediately after the baseline survey. Outcome measures will be assessed in a similarly designed household survey conducted three years after the intervention begins, before the final round of anthelminthic treatment is delivered to all villages.

Available listings of households in each village were checked and updated by the research team and VHTs to provide the sampling frame for the baseline survey, and will be further updated to provide the sampling frame for the three-year survey. For the baseline survey, a simple random sample of 45 households, expected to comprise 125 individuals, was selected from each of the study clusters. In selected and participating households, all household members (defined as people who sleep in the same house and share meals), of all ages, were eligible for inclusion in the survey.

### Outcomes

Primary outcomes for the trial, assessed at the three-year survey, are:

1. wheeze within the last 12 months (only in participants aged ≥5 years);
2. atopy: SPT positivity (only in participants aged ≥1 year); and
3. atopy: allergen-specific (as)IgE concentration (only in participants aged ≥1 year).

Secondary outcomes are:

1. visible flexural dermatitis (all participants);
2. helminth infections (all participants);
3. haemoglobin (only in participants aged ≥1 year);
4. growth as measured by height-for-age and weight-for-age z-scores (only in participants aged ≤19 years);
5. hepatosplenomegaly (all participants); and
6. vaccine responses (participants aged ≥1 year).

### Outcome measurement

Survey procedures used for baseline and planned for the three-year survey are identical. A questionnaire is completed regarding household features, and individual questionnaires are completed for each household member. Questions elicit social, demographic, and family characteristics, and information regarding asthma, eczema, and allergy symptoms. To ensure comparability with studies conducted elsewhere, we employ questions from the International Study on Allergy and Asthma in Children (ISAAC) questionnaire, with supplementary questions from the UK diagnostic criteria for atopic eczema [37,38]. The co-primary outcome, wheeze within the last 12 months, is assessed through this questionnaire.

Our pilot work suggested imperfect understanding of wheeze in this community, so the ISAAC video questionnaire for asthma, kindly provided by Dr Julian Crane (University of Otago, New Zealand), is also administered. Lung function parameters are also assessed using a hand-held spirometer (Micro 1 Diagnostic Spirometer, CareFusion, Chatham Marine, UK). This instrument has internal controls which categorise efforts as a good blow or an inadequate blow. Each participant is trained to use the spirometer, and the best result of three good blows (taken ≥20 seconds apart) is recorded (forced expiratory volume in one second (FEV_1_), forced vital capacity (FVC), and peak expiratory flow (PEF)).

A general history and examination, including height and weight, and a check for hepatosplenomegaly, is done. All individuals are examined for visible flexural dermatitis; all team members are trained in the standardised approach described by Williams [39]. SPTs are performed on all participants aged one year and older, using standard methods, with three allergens (*Dermatophagoides* mix, *Blomia tropicalis*, and German cockroach (*Blattella germanica*)) and positive and negative controls (ALK-Abelló, supplied by Laboratory Specialities (Pty) Ltd, Randburg, South Africa). Total IgE and asIgE (specific to allergens such as *Dermatophagoides* and German cockroach) are measured using enzyme-linked immunosorbent assay (ELISA) [35] (Additional file 1); concentrations are measured in ng/mL. Binary variables for an atopic response for each allergen are created by converting results to kUa/L using the ImmunoCAP conversion factor of 2.42 ng/mL to 1 kUa/L [40], and applying the European Academy of Allergy and Clinical Immunology (EAACI) recommended cut-off of 0.35 kUA/L.

Each participant is asked for one stool sample, from which two slides are examined (by different individuals) using the Kato-Katz method [41]. The remaining sample is suspended in ethanol and stored at −80°C to allow further investigation for *Necator americanus* and *Strongyloides stercoralis*, and, for a subset of 200 participants in the baseline survey, *Schistosoma mansoni* and *Ancylostoma duodenale*, using real-time polymerase chain reaction (PCR) [42,43] (Additional file 1). In addition, urine is collected for assessment of circulating cathodic antigen (CCA) of *S. mansoni* (Rapid Medical Diagnostics, Pretoria, South Africa)*.*

Blood samples of 14 mL are obtained from individuals aged ≥13 years, 10 mL from children aged 5 to 12 years, and 6 mL from children aged 1 to 4 years; no blood sample is obtained from infants. Blood samples are used for haematology, haemo-parasitology, and immunological assays. Haemoglobin is assessed by HemoCue® (HemoCue AB, Angelholm, Sweden); *Mansonella perstans* infection is determined by a modified Knott’s method [44]. Malaria is assessed by thick blood film. Adults are offered HIV counselling and testing in collaboration with health service providers.

To assess vaccine responses, assays are conducted for levels of antibody to vaccine antigen. Anti-tetanus and anti-measles antibody levels are measured by ELISA [45]. Cellular responses to tetanus toxoid and mycobacterial antigens are also measured using a whole blood assay, as previously described and used in our studies [46]. For comparison, responses to relevant allergens and schistosome antigens are also measured.

In a sample archive, peripheral blood mononuclear cells are separated and stored in liquid nitrogen. Serum, plasma, and a whole cell pellet suitable for DNA extraction are also stored. Participants’ consent provides for use of samples in future studies, subject to approval by the relevant ethical committees. Depending upon the epidemiological results obtained, these samples should allow us to investigate pathways of the immune response to allergens that are modified during helminth infection, using a range of techniques.

### Sample size

For the three-year survey we aim to sample 125 individuals per cluster. Based on preliminary data collected before the trial, prevalence of each primary outcome was estimated to be 10%. Table 1 indicates the power that the trial will have to detect risk ratios (RR) of 1.4 to 1.6 for intensive versus standard anthelminthic treatment at a 5% significance level, assuming a coefficient of variation of 0.2 [47]. If the coefficient of variation is higher, at 0.25, then the trial will have approximately 80% power to detect risk ratios of 1.5 or higher.

**Table 1:** **Power to detect differences between the two intervention arms at a significance level of 5%, assuming a coefficient of variation of 0.2**

**Outcome**	**Sample size per arm**	**Prevalence in standard intervention arm**	**Risk ratio for intervention effect**		
			**1.4**	**1.5**	**1.6**
Wheeze within last 12 months in participants ≥5-years-old	1,300	10%	67%	83%	92%
Atopy: positive skin prick test in participants ≥1-year-old	1,560	10%	72%	87%	95%
Atopy: allergen-specific immunoglobulin E concentration >0.35 kUA/L in participants ≥1-year-old	1,560	10%	72%	87%	95%

### Analysis plan

Analysis will be done by intention-to-treat, that is, participants will be analysed in the clusters in which they are resident at the three-year survey, regardless of whether they have received the intervention or not. We will employ statistical methods that allow for within-cluster correlations. Primary analysis will be done at the cluster-level since this is most robust when the number of clusters is relatively small [48].

For binary outcomes, cluster-specific proportions will be calculated, and the arithmetic mean of these within each intervention arm will be used as a summary measure of the proportion experiencing the outcome in that arm. Crude risk ratios will be calculated directly from these summary measures. *P* values for the effect of the intervention will be calculated using t-tests comparing cluster-level proportions between the treatment arms, and 95% confidence intervals for risk ratios will be derived using a Taylor series approximation approach to estimate standard error. It is possible that cluster-specific proportions will be positively skewed. If this is the case, log transformations will be applied and the analysis approach will be modified accordingly [48].

Analysis of quantitative outcomes will follow a similar approach, with the effect of the intervention quantified as the difference in mean outcome between the two arms, and a 95% confidence interval for this calculated directly using the t-distribution and standard formulae for the standard error of the difference between two means.

Adjusted analyses for the effect of the intervention will also be conducted. For each outcome, the cluster-specific prevalence (or mean for quantitative outcome) of the outcome as assessed at the baseline survey will be adjusted for, *a priori*, to improve the precision of intervention effect estimates. We will also investigate adjustment for prognostic factors that showed imbalance between treatment arms in the baseline survey, despite randomisation. Adjustment will be performed using a two-stage approach [48].

### Ethical considerations

Ethical approval has been granted from the Research and Ethics Committee of the Uganda Virus Research Institute (reference: GC127), the Uganda National Council for Science and Technology (reference: HS 1183), and the London School of Hygiene and Tropical Medicine (reference: 6187).

Study participants suffer the inconvenience and minor discomfort entailed by the study procedures, in particular the blood draws and SPTs. The research team are experienced in all these procedures, which are regularly undertaken within epidemiological and clinical studies of asthma, eczema, and atopy. SPTs are carried out by an experienced nurse and, although not painful, could be viewed as invasive; we therefore obtain a separate consent signature for this procedure.

Participants are provided with a soft drink or snack when procedures have been completed. No financial compensation is provided, since participants are not required to travel or incur costs. However, the communities benefit from anthelminthic drug provision, and individuals who are considered likely to have asthma or eczema are advised on the management of the condition. Moreover, research visits offer opportunities for Koome Health Centre staff to conduct outreach visits at which general health care and immunisations can be provided (health centres often lack funds to provide transport for these activities).

As schistosomiasis can cause significant direct morbidity [11] all villages receive at least the standard intervention. A potential concern is that standard intervention communities may be disadvantaged compared to the intensive intervention communities. However, because the VCD has been unable to reach some island communities regularly, the research programme is likely to result in better coverage throughout the study area than in the past.

Mass treatment with anthelminthics is a well-established public health practice and important adverse and serious adverse events are not expected. Higher rates of adverse events and serious adverse events are expected in the intensive arm because these villages are treated more often, and information is requested more often. This will be taken into account, for example, by reporting rates per treatment round.

## Discussion

Mass anthelminthic treatment has been widely advocated as a means of improving general health outcomes, but its benefits are not conclusively proven for these outcomes, and there is some evidence that worm removal may lead to an increase in allergy-related disease outcomes [35]. The LaVIISWA study aims to address the relative risks and benefits of mass anthelminthic treatment and to provide a platform for immunological investigation of effects observed. To effectively address the first of these objectives, randomisation was done at the village level. This cluster-randomisation approach guards against dilution of the effects of anthelminthic treatment by re-infection from untreated neighbours, is expected to result in a reduction in transmission within the whole village, and replicates programmatic approaches. The LaVIISWA study is the first cluster-randomised trial to investigate the effects of anthelminthic treatment on allergy-related outcomes in an area where *S. mansoni* is the dominant helminth species, and is also the first to examine the effect of treating the whole community. The three-year follow-up period is longer than has been used in other trials examining similar questions [18], thus longer-term effects of worm removal should be captured.

### Experiences from conducting the trial

During initial visits community members welcomed the trial, and leaders from all villages attended the randomisation ceremony and gave written consent for their village’s participation. Logistical challenges identified included the requirement for a suitable boat (a motorised transport canoe, or ‘*kinara*’, built locally) and suitable accommodation: lodges were identified in two villages, and a wooden dormitory built in a third, with the support of the local council, generating three bases from which all villages can be reached fairly comfortably within a day. Planning and equipping of a mobile field clinic and lab was also required. The team spends a week in the field at a time. Cold storage for reagents and plasma and serum samples is managed using ice boxes, with ice brought from the mainland, and topped up in the islands if necessary (ice is brought in regularly from the mainland to service the fishing industry).

Between October 2012 and July 2013, the study research team conducted the pre-intervention baseline survey and, together with VHTs, delivered the first round of the intervention. Although an up-to-date household listing was used as a sampling frame for the survey, houses were frequently found to be temporarily or permanently empty, and the average number of occupants per household was lower than expected. Of the 1,170 households (45 per cluster) selected for the baseline survey, 88% agreed to take part, and 2,316 (95%) of the 2,427 residents of these households were included in the survey. Of these, 92% gave a blood sample, 86% provided a stool sample, and 93% of those aged at least one year underwent SPT.

The concepts of wheeze and eczema are not well understood in the study setting. There is no direct translation of the word wheeze in the vernacular, Luganda, which is the *lingua franca* in the islands. The question, ‘Has your child had wheezing or whistling in the chest in the past 12 months?’ from the ISAAC questionnaire is rendered, ‘*Omwana wo yali afunyeko akakaaba mu kifuba mu bbanga lya myezi 12 egiyise*?’ which literally means, ‘Has your child had a crying sound in the chest in the past 12 months?’. To aid understanding, all participants were shown the video questionnaire for wheeze. Agreement between reported wheeze in the last 12 months and responding yes to any question in the video questionnaire was fairly low (κ = 0.38), although stronger when restricted to participants under five-years-old (κ = 0.66). Reassuringly, participants reporting wheeze had, on average, reduced FEV_1_ and PEF results (mean FEV_1_: 98.1% and 104.3% of the predicted value for age and sex for those with and without wheeze, respectively, t-test *P* = 0.03; mean PEF: 88.3% and 99.2% for those with and without wheeze, respectively, t-test *P* <0.001).

The team encountered logistical challenges in examining Kato-Katz slides within the 30 to 60 minutes necessary for reliable detection of hookworm species, and *S. stercoralis* cannot be detected by the Kato-Katz method. Therefore, stored stool samples from all participants were investigated for *N. americanus* and *S. stercoralis* using faecal PCR. For a subgroup of 200 participants, samples were also investigated using faecal PCR for *S. mansoni* and *A. duodenale* . PCR results for *S. mansoni* showed good agreement with Kato-Katz results, and no *A. duodenale* infections were detected. Thus further investigation of remaining participants for these species using PCR was not done.

### Baseline characteristics of the trial population

Baseline data were available from 2,316 residents of 1,026 households. Table 2 summarises baseline characteristics of villages and households, and Table 3 summarises characteristics of individuals, overall and by trial arm. The environment is rather uniform: accommodation generally comprises single-storey buildings of rudimentary wooden construction (95%), with a mixture of thatched and plastic sheeting roofs (90%), and beaten earth floors (99%). Sanitation is limited, with only a quarter of villages having any public toilets, and fewer than 10% of households having access to a private toilet. Only a third of villages have a water source other than the lake, with a large majority of households (75%) obtaining water for drinking and other purposes entirely from the lake. A total of 42% of the households owned animals, with chickens and pigs being the most common, and nearly all of these frequently entered the house. The population has a varied tribal background, however, with over 30 tribes reported, representing all regions of Uganda, as well as neighbouring countries in East and central Africa, only 35% are Baganda (the dominant tribe on the mainland in this district). Of the adults, 53% are fishermen or involved in work related to the fishing industry, 15% are involved in farming or lumbering, 8% are housewives, while others provide services such as shops, food, and entertainment. The age distribution of cluster residents is bimodal, with low numbers of children aged five to eighteen years, because children are sent to school on the mainland. Among adults, the sex distribution is weighted towards men.

**Table 2:** **Comparison of village and household-level characteristics, between trial arms**

**Characteristics** ^**1**^	**Standard anthelminthic treatment**	**Intensive anthelminthic treatment**
Village characteristics	(n = 13)	(n = 13)
Households per village, median (IQR)	180 (114 to 290)	265 (98 to 296)
Villages with an alternative water source to the lake	23%	38%
Villages with at least one public toilet	46%	8%
Villages where some households have privately owned toilets	69%	92%
Household characteristics	(n = 524)	(n = 502)
Crowding		
≤1 person/room	50%	44%
1-2 people/room	26%	32%
>2 people/room	24%	25%
Own animal(s)	41%	43%
Asset score, mean (SD)^2^	2.7 (1.6)	2.6 (1.6)
Wall materials		
Wood	98%	92%
Mud and wattle	2%	8%
Other	1%	0%
Roof materials		
Plastic sheets and thatch	69%	75%
Thatch	16%	17%
Iron sheets	13%	7%
Plastic sheets	2%	1%
Floor materials		
Beaten earth	49%	50%
Covered beaten earth	49%	49%
Other	2%	1%
Indoor cooking with wood/charcoal/paraffin	55%	63%
Household has toilet	7%	9%
Household takes drinking water from lake	81%	69%
Household takes washing water from lake	93%	91%
Household had no mosquito control measures	50%	48%

^1^Missing values in standard and intensive arms, respectively: crowding: 0, 1; animal ownership: 0, 1; asset score: 0, 1; house construction materials: 0, 1; indoor cooking: 0, 1; household toilet: 0, 1; household drinking water: 1, 1; household washing water: 0, 1; and household mosquito control: 0, 1. ^2^Asset score is number of items owned out of car, boat, motorbike, bicycle, television, radio, mobile phone, and bed. IQR, Interquartile range.

**Table 3:** **Comparison of individual-level characteristics, between trial arms**

**Characteristics** ^**1**^	**Standard anthelminthic treatment (n = 1,159)**	**Intensive anthelminthic treatment (n = 1,157)**
Socio-demographic characteristics		
Age in years, median (IQR)	24 (8 to 33)	24 (7 to 32)
Sex, % male	56%	53%
Occupation type (adults only)		
No personal income	11%	10%
Unskilled, no income	16%	22%
Some skill or capital required, low income	59%	46%
Education, skills or capital required, low to moderate income	5%	9%
Facility owner (such as a shop, boat, or restaurant)	8%	11%
Maternal tribe		
Central	29%	36%
Western	16%	12%
Eastern	19%	23%
Northern	17%	14%
Non-Ugandan	18%	14%
Paternal tribe		
Central	33%	40%
Western	15%	12%
Eastern	20%	21%
Northern	18%	14%
Non-Ugandan	13%	12%
Number of older siblings, mean (SD)	2.8 (2.8)	2.9 (3.1)
Number of younger siblings, mean (SD)	2.6 (2.7)	2.6 (2.7)
Any previous worm treatment in last 12 months	44%	39%
Treatment with albendazole in last 12 months	29%	27%
Treatment with praziquantel in last 12 months	20%	14%
Infections		
*Schistosoma mansoni* (Kato-Katz)	56%	49%
*Schistosoma mansoni* (urine CCA)	72%	73%
*Schistosoma mansoni* intensity (Kato-Katz)		
Uninfected	44%	51%
Low	22%	21%
Moderate	15%	13%
Heavy	18%	14%
*Hookworm (Necator americanus;* PCR)	23%	23%
*Strongyloides stercoralis* (PCR)	13%	13%
*Trichuris trichiura* (Kato-Katz)	12%	11%
*Ascaris lumbricoides* (Kato-Katz)	2%	1%
*Mansonella perstans*	3%	2%
*Plasmodium falciparum*	6%	7%
HIV (adults only)	17%	19%

^1^Missing values in standard and intensive arms respectively: age: 4, 1; sex: 6, 7; occupation: 8, 7; maternal tribe: 18, 17; paternal tribe: 15, 17; older siblings: 6, 7; younger siblings: 7, 9; any worm treatment: 17, 15; albendazole treatment: 28, 32; praziquantel treatment: 30, 31; Kato-Katz results: 172, 148; PCR results: 172, 150; urine results: 642, 757; *M. perstans*: 115, 102; *P. falciparum*: 107, 94; HIV: 65, 60. CCA, circulating cathodic antigen; IQR, interquartile range; PCR, polymerase chain reaction.

**Figure 4:**
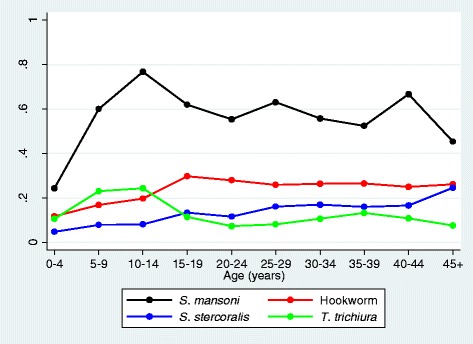
Prevalence of *Schistosoma. mansoni*, *hookworm, Strongyloides stercoralis*, and *Trichuris trichiura* infections, by age. *S. mansoni* and *T. trichiura* determined by Kato-Katz, *N. americanus*, and *S. stercoralis* determined by PCR.

Baseline findings indicate a very heavy burden of disease in the study population. Adult HIV prevalence was 18%, over twice the national figure of 7.2% [49], although asymptomatic malaria was relatively uncommon (6%). Helminth prevalence was very high despite the efforts of the national programme to provide annual treatment: 52% were infected with *S. mansoni*, approximately a third of whom were classified as having heavy infections based on WHO cut-offs [50]. Urine assay and faecal PCR detection of *S. mansoni* were done on two different subgroups of the study participants; urine assay classified 72% of individuals as infected (compared to 48% classified as infected by Kato-Katz in this subgroup), while PCR classified 56% as infected (compared to a Kato-Katz figure of 47% in the PCR-tested subgroup). Prevalence of hookworm (using PCR), *S. stercoralis* (using PCR), *T. trichiura* (using Kato-Katz), and *A. lumbricoides* (using Kato-Katz) were 23%, 13% 12%, and 1%, respectively, with an overall figure of 31% infected with an albendazole-susceptible helminth species. Figure 4 shows the prevalence of the most common helminth infections by age group. *S. mansoni* was highly prevalent in all but the youngest age group. The prevalence of *T. trichiura* peaked in school-aged children. Hookworm prevalence increased to a peak in the 15 to 19 year age group and then gradually declined, while prevalence of *S. stercoralis* increased steadily with age.

The results from the three different techniques for assessing *S. mansoni* infection status illustrate the fact that prevalence estimates based on Kato-Katz analysis of a single stool sample, an approach known to lack sensitivity [51], are likely to be underestimates. For the post-intervention outcome survey, we will conduct urine assays for all participants, and do PCR for *S. stercoralis* and hookworm. Combining these techniques will give good sensitivity for each helminth in the final survey.

Data on allergy-related primary and secondary outcomes at baseline is shown in Table 4. Reported wheeze in the last 12 months was rare (<5%), although the proportion reporting wheeze in response to the video questionnaire was higher, at 10%. Visible flexural eczema was very rare. A total of 20% of participants were atopic as assessed by SPT, with German cockroach being the most common allergen. The median (IQR) asIgE was 72 (8.5 to 199.5) ng/mL for *Dermatophagoides* and 11 (3.5 to 32.5) ng/mL for German cockroach, and 77% of participants had a *Dermatophagoides* IgE response higher than the EAACI cut-off of 0.35 kUa/L, with 87% above the cut-off for German cockroach.

**Table 4:** **Comparison of outcomes ascertained at the baseline survey, between trial arms**

**Outcomes** ^**1**^	**Standard anthelminthic treatment (n = 1,159)**	**Intensive anthelminthic treatment (n = 1,157)**
Wheeze in last 12 months	5%	4%
Video questionnaire wheeze in last 12 months	9%	10%
Atopy (skin prick test)		
Any	21%	20%
*Dermatophagoides*	10%	10%
*Blomia*	10%	10%
German cockroach	14%	13%
Atopy (allergen-specific immunoglobulin E > 0.35 kUa/L)		
*Dermatophagoides*	76%	78%
German cockroach	86%	89%
Visible flexural dermatitis	1%	1%

^1^Missing values in standard and intensive arms respectively: wheeze: 7, 8; video questionnaire: 123, 18; atopy (SPT): 165, 175; atopy (asIgE): 107, 92; visible flexural dermatitis: 100, 71.

Although some variables appeared a little unbalanced in the village-level comparison (where the denominator for each trial arm is 13), comparisons at household and individual level suggest that good balance has been achieved between trial arms.

### Potential limitations

For ethical reasons, villages in the standard arm will receive the VCD-recommended schedule of anthelminthic treatment, thus we will not be able to investigate the effects of worm removal with respect to an untreated population. However, the standard treatment is not expected to be as effective as the intensive treatment in worm removal due to the very high baseline prevalence and intensity, poor sanitation, and very limited access to water from sources other than the lake, all of which make the chances of re-infection considerable. Interim surveys of helminth prevalence will be conducted annually in each study cluster, to assess the effectiveness of each trial intervention.

The efficiency of a cluster-randomised trial with fixed total sample size is maximised with a large number of clusters of small sample size. The LaVIISWA study includes a relatively small number of clusters, due to both logistic and budgetary constraints. Nevertheless, the trial has good power to detect moderate to large effects of the intervention. A possible consequence of having a relatively small number of clusters was imbalance between trial arms, but baseline data indicate that the restricted randomisation approach was successful in achieving balance on important covariates. These data will also allow us to conduct adjusted analyses allowing for baseline variations in outcome prevalence between individual study clusters (which may exist even though overall balance between trial arms has been achieved), thus minimising residual variability in the analysis. We have presented baseline data summary statistics unadjusted for the survey design (which is not self-weighting); however, figures allowing for this are very similar.

The diagnosis of asthma within epidemiological studies is challenging because the condition is, by definition, variable and individuals may be asymptomatic at the time of assessment. Comparisons between approaches have been made, with the conclusion that standardised questionnaires about wheeze have the greatest validity for population surveys, particularly when supplemented with a video questionnaire [52,53]. For this reason we decided to use reported wheeze as the primary outcome to reflect asthma prevalence in this study. The use of pictures and videos has aided understanding of the conditions being studied and the associations between reported wheeze, video questionnaire responses, and spirometry provides some reassurance.

## Conclusions

Despite logistical challenges, the study team have successfully conducted a large baseline survey, and the interventions have now successfully been rolled out to all clusters. Apart from providing baseline data, conducting the baseline survey has provided information that will be essential in refining the approach to be used for the three-year post-intervention survey. It is possible that short-term interventions (such as the three-year duration of this trial) may have limited effects. However, the study design is such as to allow for longer-term follow-up, and repeat surveys at a greater time-interval, if our results suggest that this is likely to be of interest.

## Trial status

The cluster-level intervention was rolled out from October 2012 and will conclude in July 2016. Recruitment into the final survey where the effect of the cluster-level intervention will be assessed will begin in October 2015 and is expected to conclude in July 2016.

## Additional file

Additional file 1:
**Detailed laboratory methods for measurement of allergen-specific Immunoglobulin (Ig) E ELISA, and for helminth DNA extraction and real-time polymerase chain reaction (PCR).**

## Abbreviations

asIg: Allergen-specific immunoglobulin
CCA: Circulating cathodic antigen
EAACI: European academy of allergy and clinical immunology
ELISA: Enzyme-linked immunosorbent assay
FEV_1_: Forced expiratory volume in one second
FVC: Forced vital capacity
Ig: Immunoglobulin
IQR: Interquartile range
ISAAC: International study on allergy and asthma in children
LaVIISWA: Lake Victoria Island Intervention Study on Worms and Allergy-related diseases
PCR: Polymerase chain reaction
PEF: Peak expiratory flow
RR: Risk ratio
SPT: Skin prick test
VCD: Vector control division
VHT: Village health team

## References

[1] Bahadori K, Doyle-Waters MM, Marra C, Lynd L, Alasaly K, Swiston J (2009). Economic burden of asthma: a systematic review. BMC Pulm Med..

[2] Carroll CL, Balkrishnan R, Feldman SR, Fleischer AB, Manuel JC (2005). The burden of atopic dermatitis: impact on the patient, family, and society. Pediatr Dermatol.

[3] Masoli M, Fabian D, Holt S, Beasley R (2004). The global burden of asthma: executive summary of the GINA Dissemination Committee report. Allergy.

[4] Nathan RA (2007). The burden of allergic rhinitis. Allergy Asthma Proc.

[5] Anandan C, Nurmatov U, van Schayck OC, Sheikh A (2010). Is the prevalence of asthma declining? Systematic review of epidemiological studies. Allergy.

[6] Anderson HR, Gupta R, Strachan DP, Limb ES (2007). 50 years of asthma: UK trends from 1955 to 2004. Thorax.

[7] Cooper PJ, Rodrigues LC, Cruz AA, Barreto ML (2009). Asthma in Latin America: a public heath challenge and research opportunity. Allergy.

[8] Souza-Machado C, Souza-Machado A, Franco R, Ponte EV, Barreto ML, Rodrigues LC (2010). Rapid reduction in hospitalisations after an intervention to manage severe asthma. Eur Respir J.

[9] The Global Asthma Report 2011. International Union Against Tuberculosis and Lung Disease, Paris, France.

[10] Hotez PJ, Brindley PJ, Bethony JM, King CH, Pearce EJ, Jacobson J (2008). Helminth infections: the great neglected tropical diseases. J Clin Invest.

[11] King CH, Dangerfield-Cha M (2008). The unacknowledged impact of chronic schistosomiasis. Chronic Illn.

[12] Kabatereine NB, Brooker S, Koukounari A, Kazibwe F, Tukahebwa EM, Fleming FM (2007). Impact of a national helminth control programme on infection and morbidity in Ugandan schoolchildren. Bull World Health Organ.

[13] Hewitson JP, Grainger JR, Maizels RM (2009). Helminth immunoregulation: the role of parasite secreted proteins in modulating host immunity. Mol Biochem Parasitol.

[14] van Riet E, Hartgers FC, Yazdanbakhsh M (2007). Chronic helminth infections induce immunomodulation: consequences and mechanisms. Immunobiology.

[15] Bentwich Z, Kalinkovich A, Weisman Z, Borkow G, Beyers N, Beyers AD (1999). Can eradication of helminthic infections change the face of AIDS and tuberculosis?. Immunol Today.

[16] Fitzsimmons CM, Dunne DW (2009). Survival of the fittest: allergology or parasitology?. Trends Parasitol.

[17] Smits HH, Everts B, Hartgers FC, Yazdanbakhsh M (2010). Chronic helminth infections protect against allergic diseases by active regulatory processes. Curr Allergy Asthma Rep.

[18] Wammes LJ, Mpairwe H, Elliott AM, Yazdanbakhsh M (2014). Global worming or deworming? A review of contrasting scientific directions. Lancet Infect Dis.

[19] Kouriba B, Chevillard C, Bream JH, Argiro L, Dessein H, Arnaud V (2005). Analysis of the 5q31-q33 locus shows an association between IL13-1055C/T IL-13-591A/G polymorphisms and *Schistosoma haematobium* infections. J Immunol.

[20] Moller M, Gravenor MB, Roberts SE, Sun D, Gao P, Hopkin JM (2007). Genetic haplotypes of Th-2 immune signalling link allergy to enhanced protection to parasitic worms. Hum Mol Genet.

[21] Lynch NR, Hagel I, Perez M, Di Prisco MC, Lopez R, Alvarez N (1993). Effect of anthelmintic treatment on the allergic reactivity of children in a tropical slum. J Allergy Clin Immunol.

[22] van den Biggelaar AH, Rodrigues LC, van Ree R, van der Zee JS, Hoeksma-Kruize YC, Souverijn JH (2004). Long-term treatment of intestinal helminths increases mite skin-test reactivity in Gabonese schoolchildren. J Infect Dis.

[23] Flohr C, Tuyen LN, Quinnell RJ, Lewis S, Minh TT, Campbell J (2010). Reduced helminth burden increases allergen skin sensitization but not clinical allergy: a randomized, double-blind, placebo-controlled trial in Vietnam. Clin Exp Allergy.

[24] Cooper PJ, Chico ME, Vaca MG, Moncayo AL, Bland JM, Mafla E (2006). Effect of albendazole treatments on the prevalence of atopy in children living in communities endemic for geohelminth parasites: a cluster-randomised trial. Lancet.

[25] Flohr C, Quinnell RJ, Britton J (2009). Do helminth parasites protect against atopy and allergic disease?. Clin Exp Allergy.

[26] World Health Organization (2002). Prevention and control of schistosomiasis and soil-transmitted helminthiasis: report of a WHO expert committee.

[27] Taylor-Robinson D, Jones A, Garner P (2009). Does deworming improve growth and school performance in children?. PLoS Negl Trop Dis.

[28] Taylor-Robinson DC, Jones AP, Garner P (2007). Deworming drugs for treating soil-transmitted intestinal worms in children: effects on growth and school performance. Cochrane Database Syst Rev..

[29] Montresor A, Engels D, Chitsulo L, Bundy DA, Brooker S, Savioli L (2001). Development and validation of a ‘tablet pole’ for the administration of praziquantel in sub-Saharan Africa. Trans R Soc Trop Med Hyg.

[30] Sousa-Figueiredo JC, Day M, Betson M, Kabatereine NB, Stothard JR (2010). An inclusive dose pole for treatment of schistosomiasis in infants and preschool children with praziquantel. Trans R Soc Trop Med Hyg.

[31] Stothard JR, Sousa-Figueiredo JC, Betson M, Green HK, Seto EY, Garba A (2011). Closing the praziquantel treatment gap: new steps in epidemiological monitoring and control of schistosomiasis in African infants and preschool-aged children. Parasitology.

[32] Speich B, Ame SM, Ali SM, Alles R, Hattendorf J, Utzinger J (2012). Efficacy and safety of nitazoxanide, albendazole, and nitazoxanide-albendazole against *Trichuris trichiura* infection: a randomized controlled trial. PLoS Negl Trop Dis.

[33] Steinmann P, Utzinger J, Du ZW, Jiang JY, Chen JX, Hattendorf J (2011). Efficacy of single-dose and triple-dose albendazole and mebendazole against soil-transmitted helminths and *Taenia* spp.: a randomized controlled trial. PLoS One.

[34] World Health Organization (1994). Report of the WHO informal consultation on hookworm infection and anaemia in girls and women.

[35] Mpairwe H, Webb EL, Muhangi L, Ndibazza J, Akishule D, Nampijja M (2011). Anthelminthic treatment during pregnancy is associated with increased risk of infantile eczema: randomised-controlled trial results. Pediatr Allergy Immunol.

[36] Ndibazza J, Muhangi L, Akishule D, Kiggundu M, Ameke C, Oweka J (2010). Effects of deworming during pregnancy on maternal and perinatal outcomes in Entebbe, Uganda: a randomized controlled trial. Clin Infect Dis.

[37] Ellwood P, Asher M, Beasley R, Clayton T, Stewart A, on behalf of the ISAAC Steering Committee and the ISAAC Phase Three Study Group (2000). ISAAC International Data Centre.

[38] Brenninkmeijer EE, Schram ME, Leeflang MM, Bos JD, Spuls PI (2008). Diagnostic criteria for atopic dermatitis: a systematic review. Br J Dermatol.

[39] Williams HC. So how do I define Atopic Eczema? A practical manual for researchers wishing to define atopic eczema. Nottingham: Dermato-Epidemiology Unit, Queens Medical Centre.

[40] Cox L, Williams B, Sicherer S, Oppenheimer J, Sher L, Hamilton R (2008). Pearls and pitfalls of allergy diagnostic testing: report from the American College of Allergy, Asthma and Immunology/American Academy of Allergy, Asthma and Immunology Specific IgE Test Task Force. Ann Allergy Asthma Immunol.

[41] Katz N, Chaves A, Pellegrino J (1972). A simple device for quantitative stool thick-smear technique in *Schistosomiasis mansoni*. Rev Inst Med Trop Sao Paulo.

[42] Verweij JJ, Brienen EA, Ziem J, Yelifari L, Polderman AM, Van Lieshout L (2007). Simultaneous detection and quantification of *Ancylostoma duodenale*, *Necator americanus*, and *Oesophagostomum bifurcum* in fecal samples using multiplex real-time PCR. Am J Trop Med Hyg.

[43] Verweij JJ, Canales M, Polman K, Ziem J, Brienen EA, Polderman AM (2009). Molecular diagnosis of *Strongyloides stercoralis* in faecal samples using real-time PCR. Trans R Soc Trop Med Hyg.

[44] Melrose WD, Turner PF, Pisters P, Turner B (2000). An improved Knott's concentration test for the detection of microfilariae. Trans R Soc Trop Med Hyg.

[45] Webb EL, Mawa PA, Ndibazza J, Kizito D, Namatovu A, Kyosiimire-Lugemwa J (2011). Effect of single-dose anthelmintic treatment during pregnancy on an infan’'s response to immunisation and on susceptibility to infectious diseases in infancy: a randomised, double-blind, placebo-controlled trial. Lancet.

[46] Elliott AM, Mawa PA, Webb EL, Nampijja M, Lyadda N, Bukusuba J (2010). Effects of maternal and infant co-infections, and of maternal immunisation, on the infant response to BCG and tetanus immunisation. Vaccine.

[47] Hayes RJ, Bennett S (1999). Simple sample size calculation for cluster-randomized trials. Int J Epidemiol.

[48] Hayes R, Moulton LH (2009). Cluster randomised trials.

[49] Joint United Nations Programme on HIV/AIDS (2013). Global report: UNAIDS report on the global AIDS epidemic.

[50] World Health Organization (1999). Monitoring helminth control programmes. Guidelines for monitoring the impact of control programmes aimed at reducing morbidity caused by soil-transmitted helminths and schistosomes, with particular reference to school-age children.

[51] Utzinger J, Booth M, N'Goran EK, Muller I, Tanner M, Lengeler C (2001). Relative contribution of day-to-day and intra-specimen variation in faecal egg counts of *Schistosoma mansoni* before and after treatment with praziquantel. Parasitology.

[52] Pearce N, Beasley R, Pekkanen J (2000). Role of bronchial responsiveness testing in asthma prevalence surveys. Thorax.

[53] Pekkanen J, Pearce N (1999). Defining asthma in epidemiological studies. Eur Respir J.

