# Supplementary material for: The Lake Victoria island intervention study on worms and allergy-related diseases (LaVIISWA): study protocol for a randomised controlled trial
Source: Trials. 2015 Apr 23;16:187. doi: 10.1186/s13063-015-0702-5 (PMC4413531; doi:10.1186/s13063-015-0702-5)
Supplement: Additional file 1: — Detailed laboratory methods for measurement of allergen-specific Immunoglobulin (Ig) E ELISA, and for helminth DNA extraction and real-time polymerase chain reaction (PCR). [file 13063_2015_702_MOESM1_ESM.docx]

**Allergen-specific Immunoglobulin (Ig) E ELISA.**

96-well microtitre plates (microlon Greiner bio-one Ltd, UK) were coated with 50 µl/well of dustmite (*Dermatophagoides pteronyssinus*) or cockroach (*Blattella germanica*) antigen (Greer Labs, Lenoir, NC, USA) diluted in coating buffer (20mM sodium bicarbonate/27mM sodium carbonate pH 9.6) at 5µg/ml concentration, incubated overnight at 4^o^C. Plates were then washed four times with phosphate-buffered saline (PBS) - tween (1X PBS + 0.03% Tween 20) and blocked with 1% w/v dried milk powder in PBS + 0.05% Tween 20 for 2hrs at room temperature. Plasma samples diluted 1/20 in assay buffer (10% fetal bovine serum in PBS + 0.05% Tween 20^o^C, standards and the blank (assay buffer) were then added in duplicate and incubated overnight at 4^o^C. To generate dustmite standards, dustmite specific IgE positive samples were pooled and quantified using Human IgE Myeloma protein (cat no. 401152, calbiochem). Seven threefold serial dilutions of the pooled positive sample were made and used to generate the standard curve. To generate cockroach standards, cockroach specific IgE positive samples were pooled and quantified as above. Similar dilutions as those done for dustmite pool were made to generate the standard curve. Plates were then washed six times and 50µl of 0.5µg/ml biotinylated monoclonal mouse anti- human IgE (Cat 555858; BD biosciences) added and incubated overnight at 4^o^C. After washing plates six times, 50µl of 1/3000 streptavidin-conjugated polyclonal antibody-Horseradish Peroxidase (Mast Group Ltd, Bootle, UK) was added and incubated 1hr at room temperature. Sigmafast o-phenylenediamine dihydrochloride (OPD)(P9187, Sigma life sciences), diluted in 0.05M phosphate-citrate buffer, pH 5.0 according to manufacturer’s instructions was added, followed by 20 minutes incubation at room temperature in the dark for development. 24µl of 2M sulphuric acid was used to stop the reaction. Plates were read at 490 and 630 nm dual wave length using an ELISA reader. Results were interpolated from standard curves using a five-parameter curve fit using Gen5 data collection and analysis software (BioTek Instruments Inc, Vermont, Winooski, USA.

**DNA extraction and real time polymerase chain reaction (PCR)**

Helminth DNA from stored stool (suspended in ethanol and frozen at -80^o^C), was extracted using a QIAamp DNA Mini Kit (cat no 51306, Qiagen) with minor changes to manufacturer`s instruction as detailed below. Samples were left at room temperature to thaw before vortexing for 5 seconds, to homogenize the ethanol-faeces mixture. 0.5ml of the homogenized mixture was transferred into an eppendorf tube and centrifuged at 13,000rpm for 3 minutes to get rid of the ethanol. The pellet was re-suspend in 200 μl of PBS with 2% polyvinylpolypyrolidone (777627, Fluka analytical, Sigma-Aldrich) and frozen overnight at -20 °C. Subsequently, the faeces-suspension was heated at 100 °C for 10 minutes; 200µl of ATL and proteinase K mixture (9 to 1 ratio) added. Samples were vortexed and incubated overnight at 55 °C in heat block. 400 μl AL Buffer was then added and the sample incubated at 70 °C for 10 minutes. This was followed by centrifugation for 1min at 13000rpm and transfer of the supernatant to 400 μl of ethanol (96-100 %). The DNA was then purified in QIAamp spin columns using consecutive washes with 500µl AW1 and 500µl AW2 buffers. 200µl AE buffer was used to elute the DNA which was then quantified on a Nano drop 2000c and diluted to 50ng/µl. Specific forward and reverse primers and Taqman probes were used in a multiplex PCR to detect the different worm DNA simultaneously. *Strongyloides stercoralis* was detected using Stro18S-1530F, Stro18S-1630R and Stro18S-1586T with Quasar705 and (BHQ2), *Necator americanus,* using Na58F, Na158R and Na81MGB with FAM and (BHQ1), *Ancylostoma duodenale*- Ad125F, Ad195R and Ad155-XS-YY with Yakima Yellow and (BHQ1) while *Schistosoma mansoni* was detected using Ssp48F, Ssp124R and Ssp78T-TR with Texas Red and (BHQ2). Phocin herpes virus DNA, extracted from the Phocin herpes virus (kindly provided by Dr. Martin Schutten, Erasmus Medical Center, Rotterdam, the Netherlands) was included in the PCR master mix thus distributed to all reaction wells as an internal control to check for PCR inhibition and proper interpretation of results. PhHV-267s, PhHVas and PhHV-305tq with Cy5 and (BHQ2) were used for Phocin herpes virus DNA detection. Serial dilutions of a positive pool were included on the plate for every run to allow setting a Ct value cut off for the test samples. The positive pool was made up of a mixture of DNA from samples *(among the study samples)* that were highly positive for *S. mansoni* and *N. americanus on* Kato-Katz, and DNA positive for *S. stercoralis* and *A. duodenale* (kindly provided by Dr. Jaco J. Verweij, St. Elisabeth Hospital, Tilburg, the Netherlands). The amplification conditions were 10 minutes at 95^o^C, 50 cycles of 15 seconds (s) at 95^o^C, 30s at 60^o^C and 30s at 72^o^C. DNA amplification, detection and data analysis were attained with the BIORAD CFX96 Real time system and Bio-Rad CFX manager Version 1.6.541.1028.
